# Supplementary material for: A potential patient stratification biomarker for Parkinson´s disease based on LRRK2 kinase-mediated centrosomal alterations in peripheral blood-derived cells
Source: NPJ Parkinsons Dis. 2024 Jan 8;10:12. doi: 10.1038/s41531-023-00624-8 (PMC10774440; doi:10.1038/s41531-023-00624-8)
Supplement: Supplementary file 1 — Supplemental Material [file 41531_2023_624_MOESM1_ESM.pdf]

## Supplementary Material

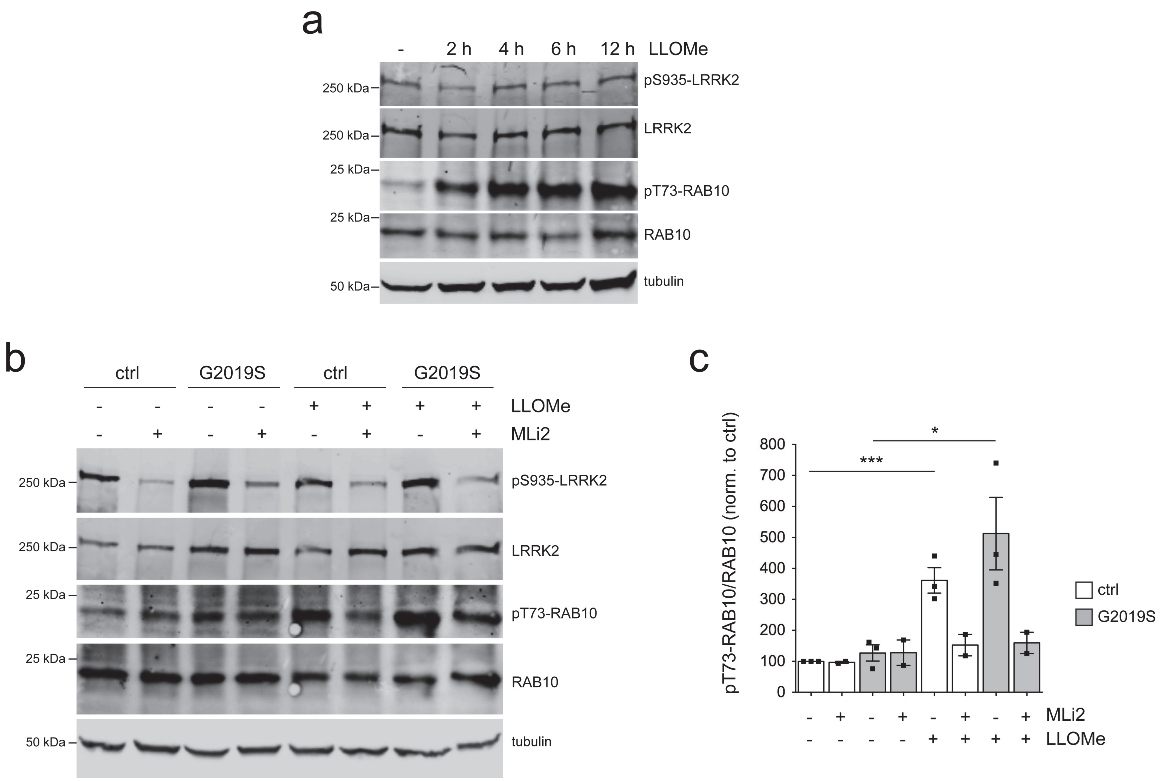


**Supplementary Figure 1. LLOMe treatment increases LRRK2-mediated pT73-Rab10 levels.** (**a**) A control LCL line was treated with LLOMe (1 mM) for increasing amounts of time as indicated, and cell extracts were subjected to multiplexed immunoblot analysis. (**b**) A control and a *G2019S-LRRK2* LCL line were treated with LLOMe (1 mM) in either the absence or presence of MLi2 (50 nM) for 2 h as indicated before immunoblot analysis. (**c**) Quantification of pT73-Rab10/Rab10 levels from the type of experiments indicated in (b). Bars represent mean ± s.e.m. (n=3 independent experiments); ctrl versus ctrl + LLOMe ($p = 0.003$), G2019S mutation versus G2019S mutation + LLOMe ($p = 0.032$). ***$p < 0.005$; *$p < 0.05$.

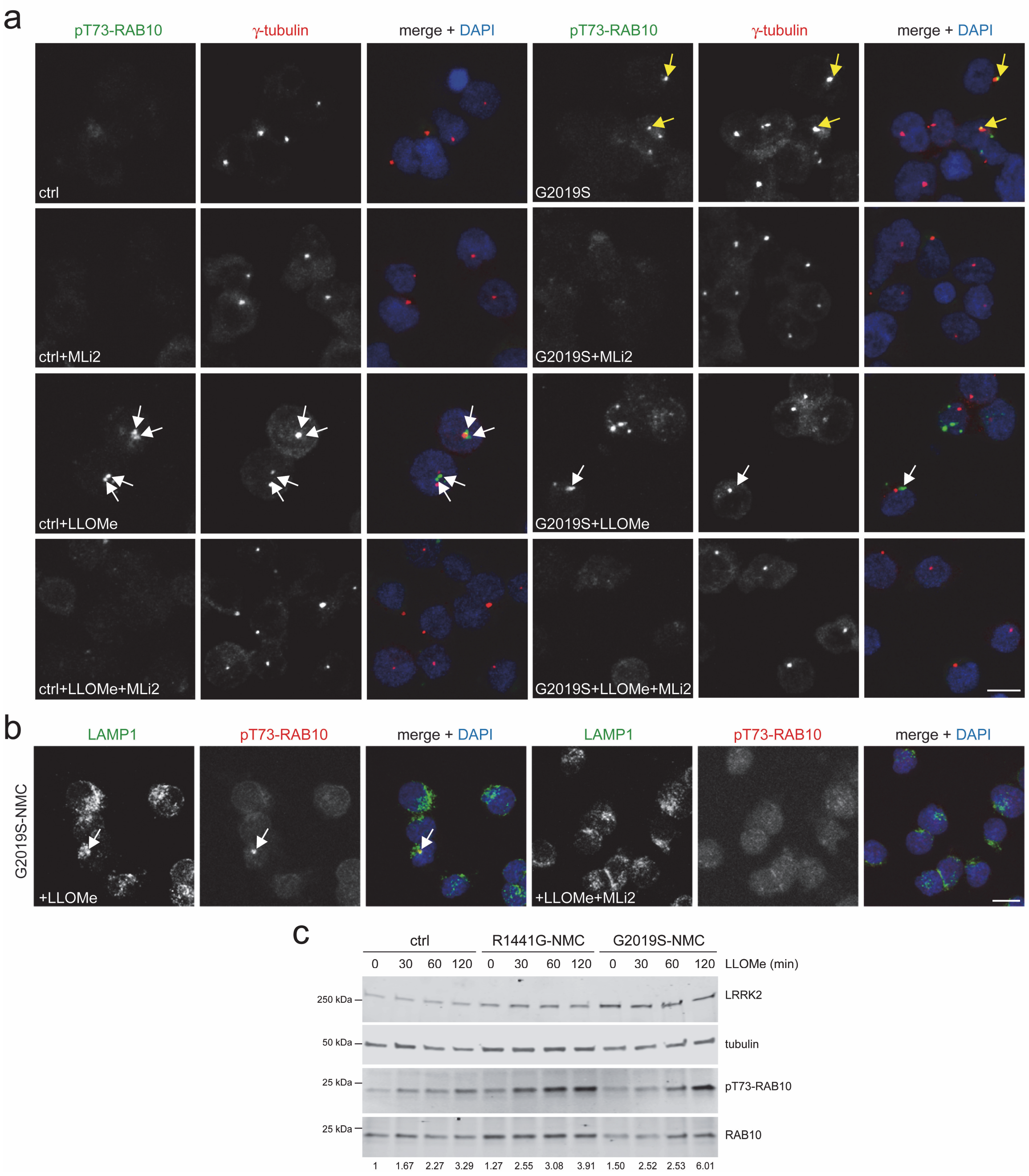


**Supplementary Figure 2. LLOMe treatment causes accumulation of pT73-Rab10 near centrosomes.** (**a**) Examples of healthy control (ctrl) or *G2019S-LRRK2* PD LCLs (G2019S) with or without treatment with LLOMe (1 mM) and MLi2 (50 nM) for 2 h as indicated, followed by staining with pT73-Rab10 antibody, γ-tubulin antibody and DAPI. Control cells display virtually no pT73-Rab10 staining. LLOMe treatment reveals dot-like pRab10 staining in some cells which is localized near the centrosome or in-between

duplicated split centrosomes (white arrows). *G2019S-LRRK2* LCLs display pT73-Rab10 staining which is overlapping with a centrosomal marker in some cells (yellow arrows). LLOMe treatment reveals dot-like pT73-Rab10 staining near the centrosome in some *G2019S-LRRK2* cells (white arrows) similar to that observed in LLOMe-treated control cells. MLi2 treatment abolishes pT73-Rab10 staining in all cases. Scale bar, 10 μm. (**b**) Example of LCL line from *G2019S-LRRK2* non-manifesting carrier (NMC) treated with LLOMe (1 mM) and with or without MLi2 (50 nM) for 2 h, followed by staining with pT73-Rab10 antibody, LAMP1 antibody and DAPI. Structure positive for pT73-Rab10 also stains positive for LAMP1. Scale bar, 10 μm. (**c**) LCL lines as indicated were treated with LLOMe (1 mM) for increasing amounts of time, and cell extracts were subjected to multiplexed immunoblot analysis.

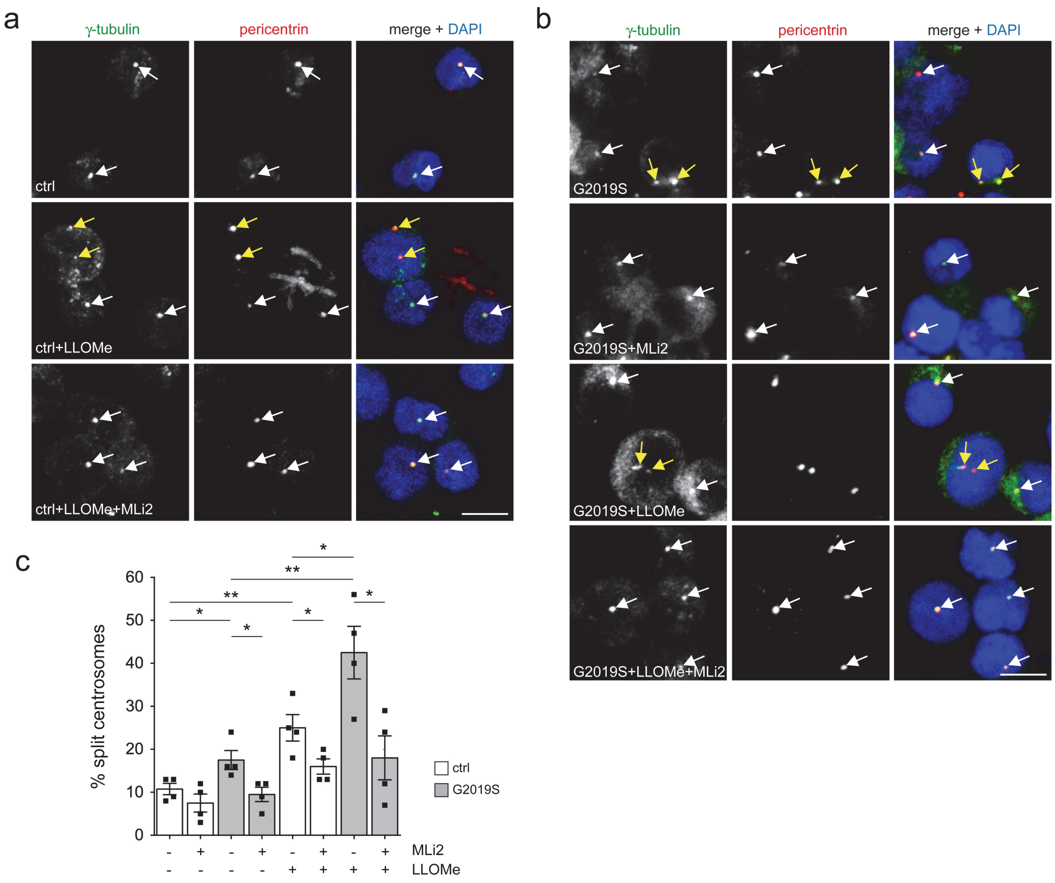


**Supplementary Figure 3. LLOMe treatment causes a LRRK2-mediated centrosomal cohesion deficit.** (**a**) Examples of healthy control (ctrl) LCLs with or without treatment with LLOMe (1 mM) and MLi2 (50 nM) for 2 h as indicated, followed by staining with two centrosomal markers (γ-tubulin and pericentrin) and DAPI. Scale bar, 10 μm. White arrows point to centrosomes, yellow arrows point to duplicated split centrosomes. (**b**) Same as (a), but *G2019S-LRRK2* PD LCLs. (**c**) Quantification of the centrosome phenotype from control and *G2019S-LRRK2* PD LCLs treated as indicated. Bars represent mean ± s.e.m. (n=4 independent experiments); ctrl versus G2019S mutation ($p = 0.039$); ctrl versus ctrl + LLOMe ($p = 0.005$); G2019S mutation versus G2019S mutation + LLOMe ($p = 0.008$); ctrl + LLOMe versus G2019S mutation + LLOMe ($p = 0.043$); G2019S mutation versus G2019S mutation + MLi2 ($p = 0.027$); ctrl + LLOMe versus ctrl + LLOMe + MLi2 ($p = 0.044$); G2019S mutation + LLOMe versus G2019S mutation + LLOMe + MLi2 ($p = 0.021$); $^{**}p < 0.01$; $^{*}p < 0.05$.

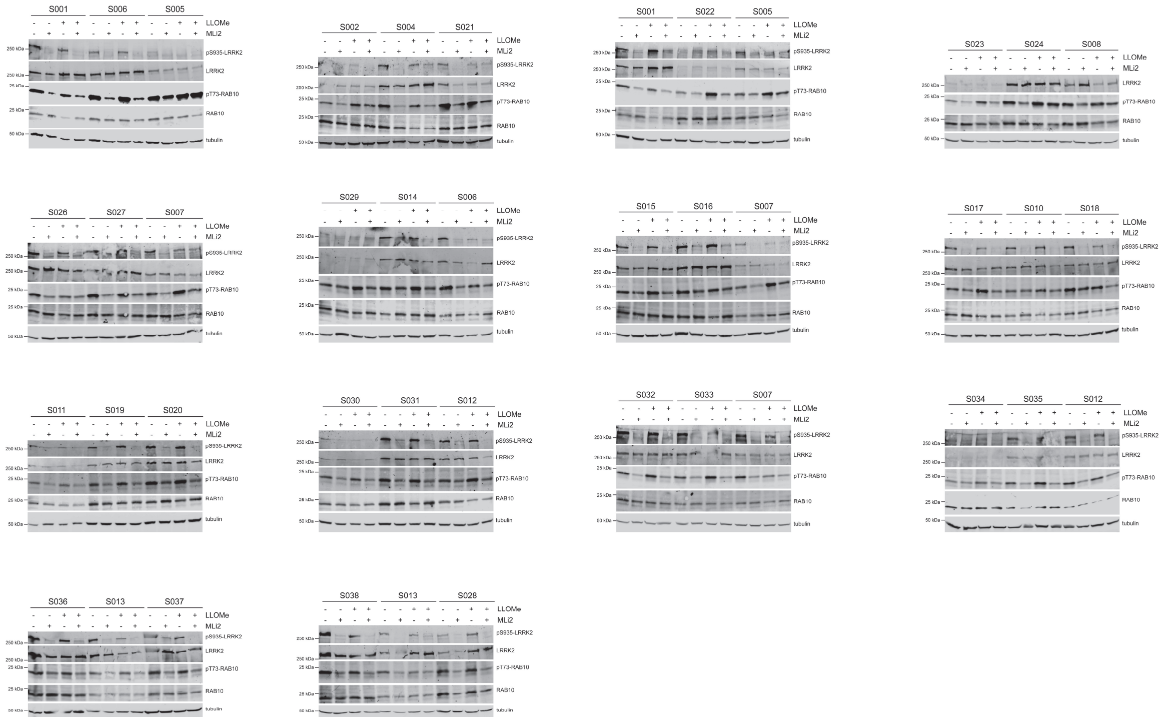


**Supplementary Figure 4. LCLs multiplexed quantitative immunoblot analysis for LLOMe-mediated and MLi2-sensitive changes in pT73-Rab10 phosphorylation.** LCLs were treated with or without LLOMe (1 mM) and with either DMSO or MLi2 (50 nM) for 2 h as indicated prior to cell lysis. Fifteen µg of extracts were loaded and subjected to quantitative immunoblot analysis with the indicated antibodies, and membranes were developed using the Odyssey CLx scan Western blot Imaging system. pT73-Rab10 and total Rab10, as well as S935-LRRK2 and total LRRK2 antibodies were multiplexed. For each gel and cell line, the values of pT73-Rab10/Rab10 in the presence of LLOMe were then normalized to those in the absence of LLOMe to obtain the % LLOMe-triggered increase in pRab10/Rab10 levels. Each line was processed in this manner 2-3 independent times.

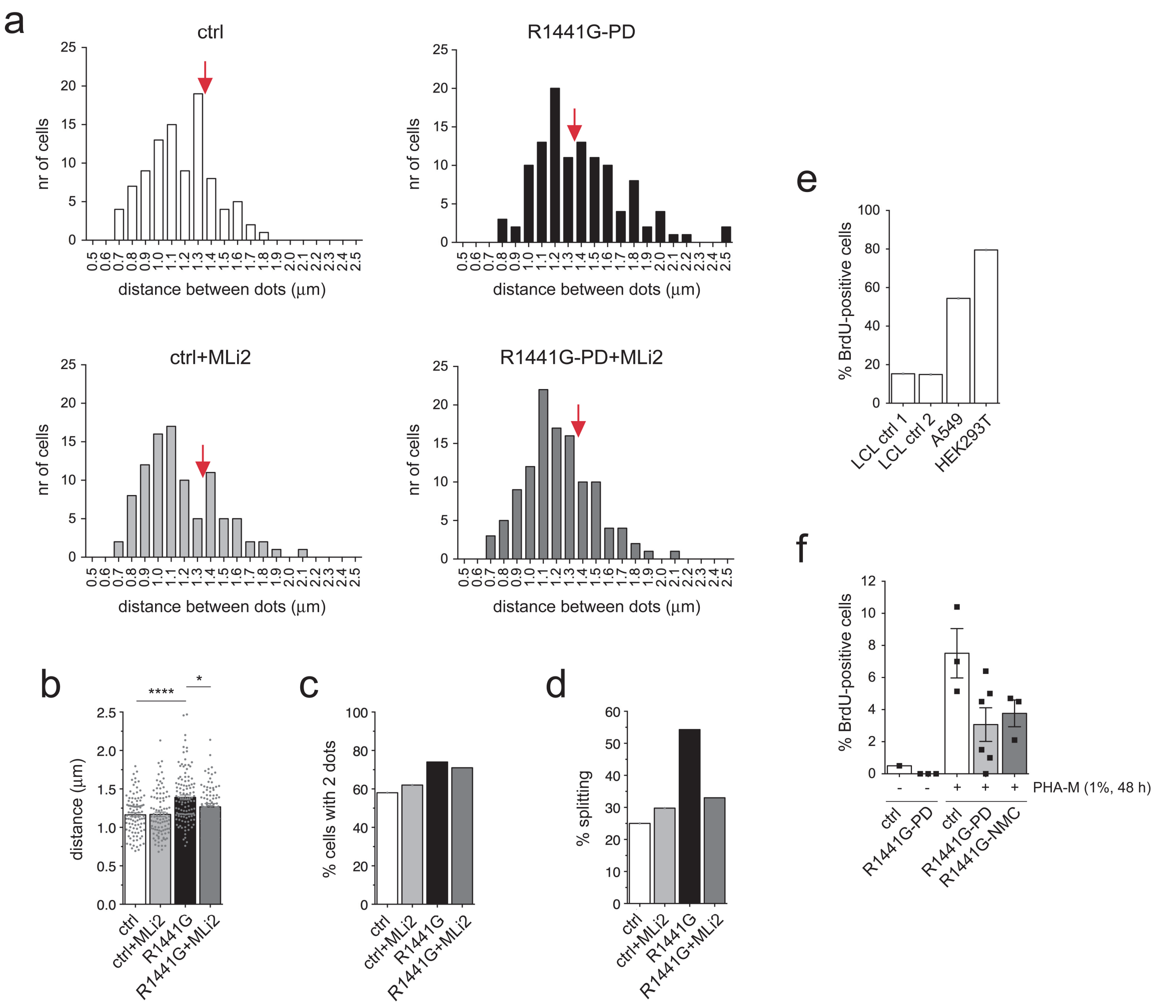


**Supplementary Figure 5. Cohesion analysis of pericentrin-positive dots from control and *R1441G-LRRK2* PD LCLs.** (**a**) Frequency histogram distribution of the number of healthy control (left) and *R1441G-LRRK2* PD LCLs (right) displaying two pericentrin-positive dots in either the absence or presence of MLi2 (50 nM, 2 h) within binned distances as indicated. For each condition, around 100-120 cells displaying 2 pericentrin dots were quantified. (**b**) Mean distances between two pericentrin-positive dots in control and *R1441G-LRRK2* PD LCLs with or without MLi2 treatment. Ctrl versus R1441G mutation ($p < 0.001$); R1441G mutation versus R1441G mutation + MLi2 ($p = 0.021$). ****$p < 0.001$; *$p < 0.05$. (**c**) Percentage of cells displaying 2 pericentrin-positive dots in control and *R1441G-LRRK2* PD LCLs with or without MLi2 treatment. (**d**) Based on the frequency distribution shown in (a), cells were scored as having a C/C split phenotype when the distances between the two pericentrin-positive dots was > 1.3 µm (red arrow),

and % splitting is indicated for control and *R1441G-LRRK2* PD LCLs with or without MLi2 treatment. (**e**) Two healthy control LCL lines, as well as wildtype A549 or HEK293T cells were treated with bromodeoxyuridine (BrdU)-containing media for 24 h before immunocytochemistry with an FITC-coupled anti-BrdU antibody and DAPI. BrdU-positive nuclei were scored from 200 cells each. (**f**) One healthy control (ctrl) and three *R1441G-LRRK2* PD PBMCs were labeled with BrdU-containing media for 24 h. Alternatively, three healthy controls, 6 *R1441G-LRRK2* PD and 3 *R1441G-LRRK2* NMC PBMCs were incubated in media containing the growth-enhancing mitogenic phytohemagglutinin-M (PHA-M, 1% v/v) for 48 h, with the last 24 h containing BrdU. Immunocytochemistry was performed as above, and BrdU-positive nuclei scored from 200 lymphocytes each. Bars represent mean ± s.e.m.

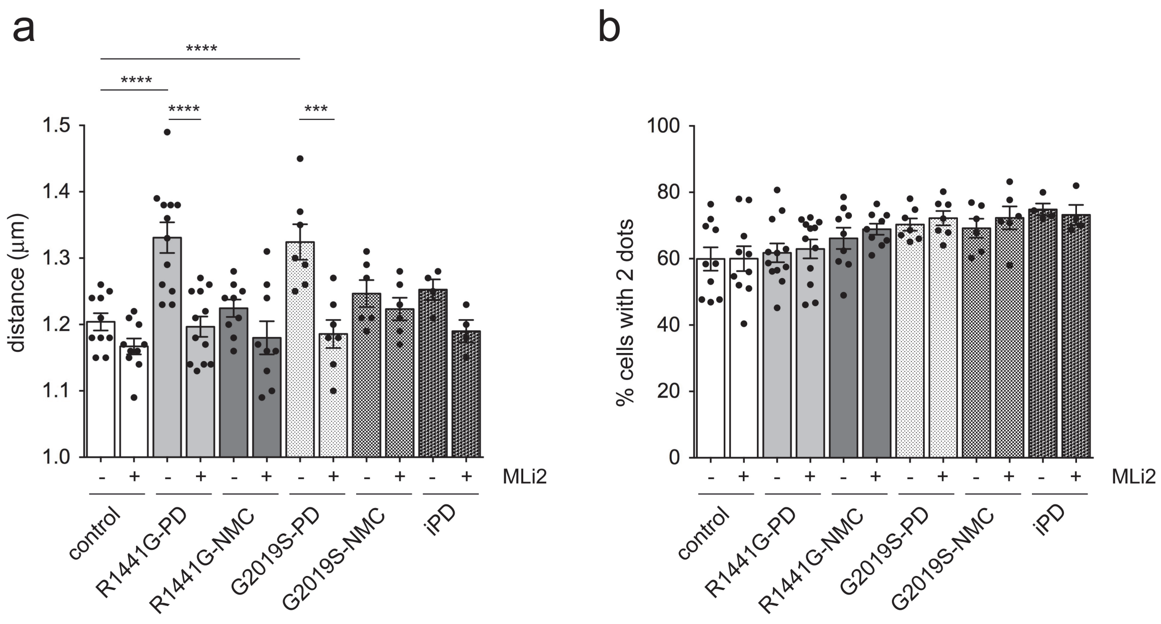


**Supplementary Figure 6. Percentage of cells with two pericentrin-positive dots and mean distances between dots in *R1441G-LRRK2* and *G2019S-LRRK2* LCLs.** (**a**) Mean distance between the two pericentrin-positive dots from the different LCL lines. Bars represent mean ± s.e.m.; ctrl versus R1441G mutation ($p = 0.0002$); ctrl versus G2019S mutation ($p = 0.0004$); R1441G mutation versus R1441G mutation + MLi2 ($p < 0.0001$); G2019S mutation versus G2019S mutation + MLi2 ($p = 0.0015$). ****$p < 0.001$; ***$p < 0.005$. (**b**) Quantification of the percentage of cells displaying two pericentrin-positive dots from a total of 100-150 cells per LCL line. Bars represent mean ± s.e.m.

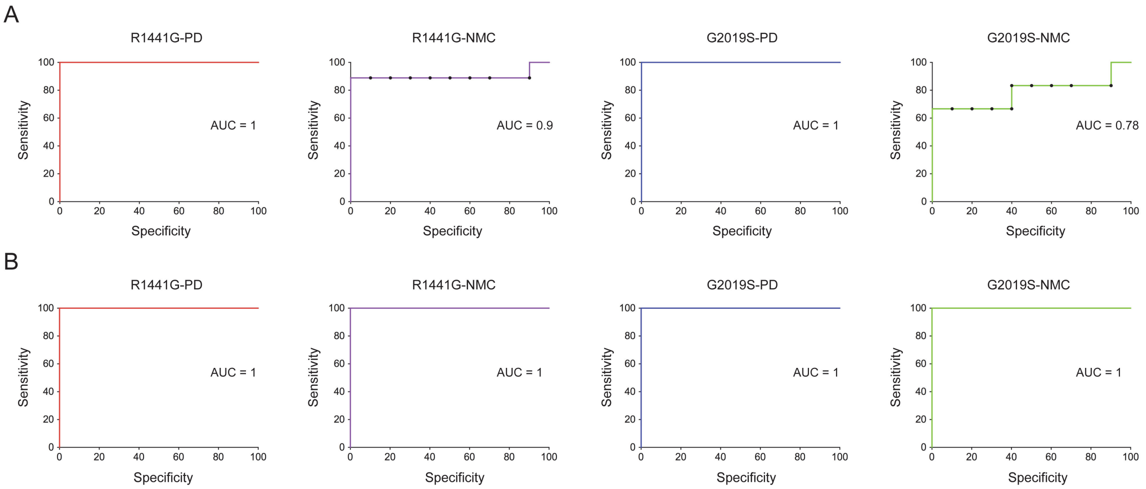


**Supplementary Figure 7. C/C cohesion phenotype and prediction AUC values for *LRRK2* mutation PD.** (**a**) ROC curves showing prediction success for PD diagnosis in LCLs from *R1441G-LRRK2* PD, *R1441G-LRRK2* NMC, *G2019S-LRRK2* PD and *G2019S-LRRK2* NMC. (**b**) As in (A), but ROC curves for prediction success in PBMC lymphocytes.

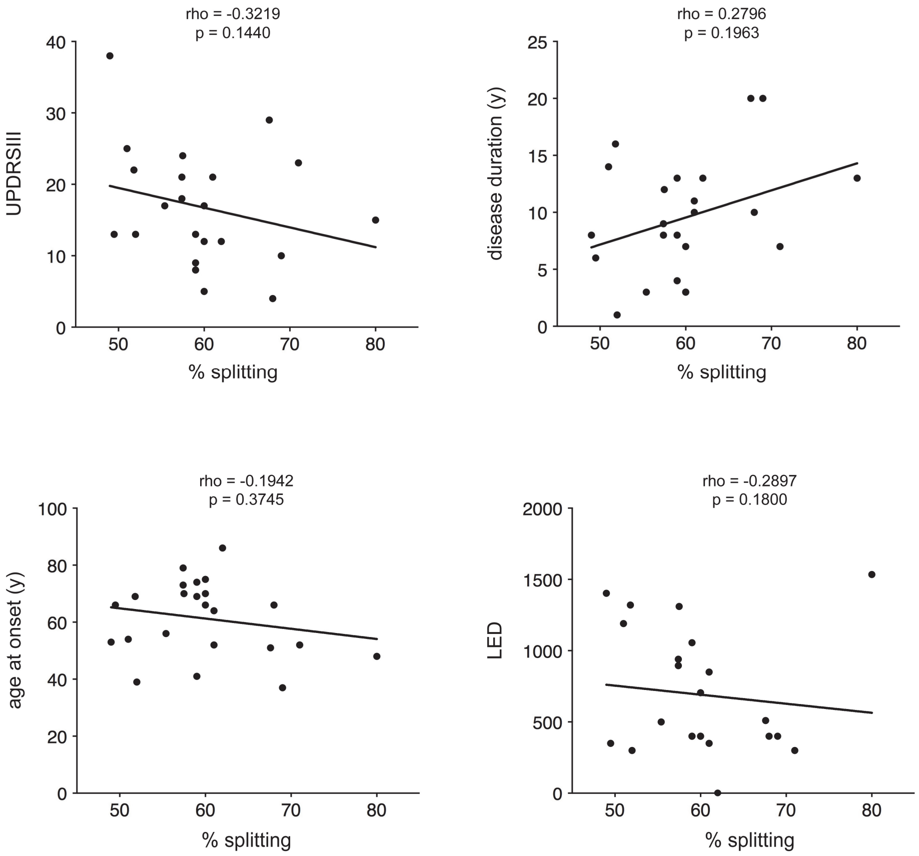


**Supplementary Figure 8. Correlation analysis between splitting phenotype and various PD clinical variables.** Spearman correlation analysis between splitting levels and UPDRSIII score, disease duration, age at onest (years) or calculated L-dopa-equivalent dose (LED) as indicated.

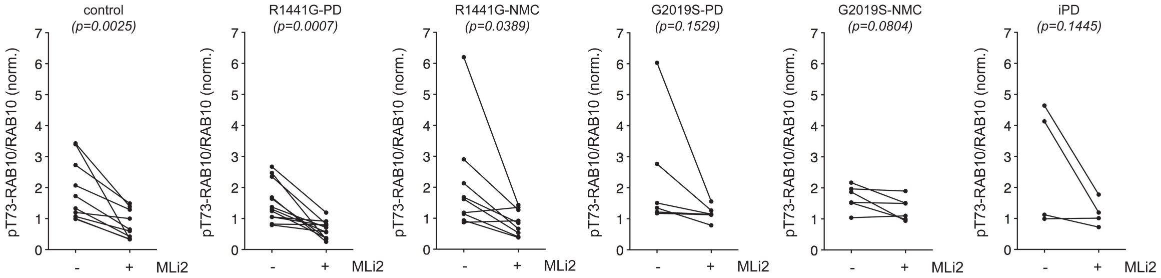


**Supplementary Figure 9. Analysis of MLi2-mediated alterations in pT73-Rab10/Rab10 levels in *R1441G-LRRK2* and *G2019S-LRRK2* LCLs.** Paired t-test analysis of pT73-Rab10/Rab10 levels from each cell line in the absence or presence of MLi2. Note that the pT73-Rab10/Rab10 levels are reduced by MLi2 treatment in most cell lines.

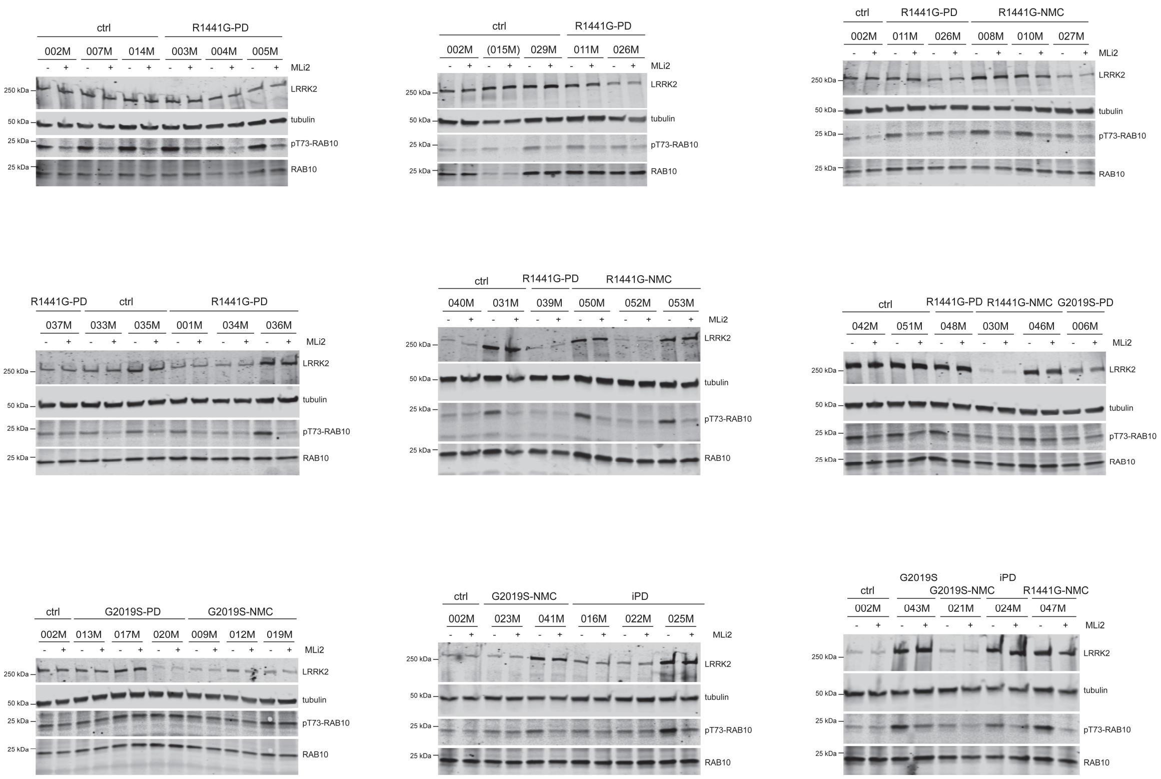


**Supplementary Figure 10. Multiplexed quantitative immunoblot analysis of LCLs.** LCLs were treated with or without MLi2 (50 nM) for 2 h as indicated prior to cell lysis. Fifteen μg of extracts were loaded and subjected to quantitative immunoblot analysis with the indicated antibodies, and membranes were developed using the Odyssey CLx scan Western blot Imaging system. pT73-Rab10 and total Rab10 antibodies were multiplexed. For each gel and cell line, the values of pT73-Rab10/Rab10 were then normalized to an internal standard (control line 002M) to compare samples run on different gels. Each line was processed in this manner 2-3 independent times.

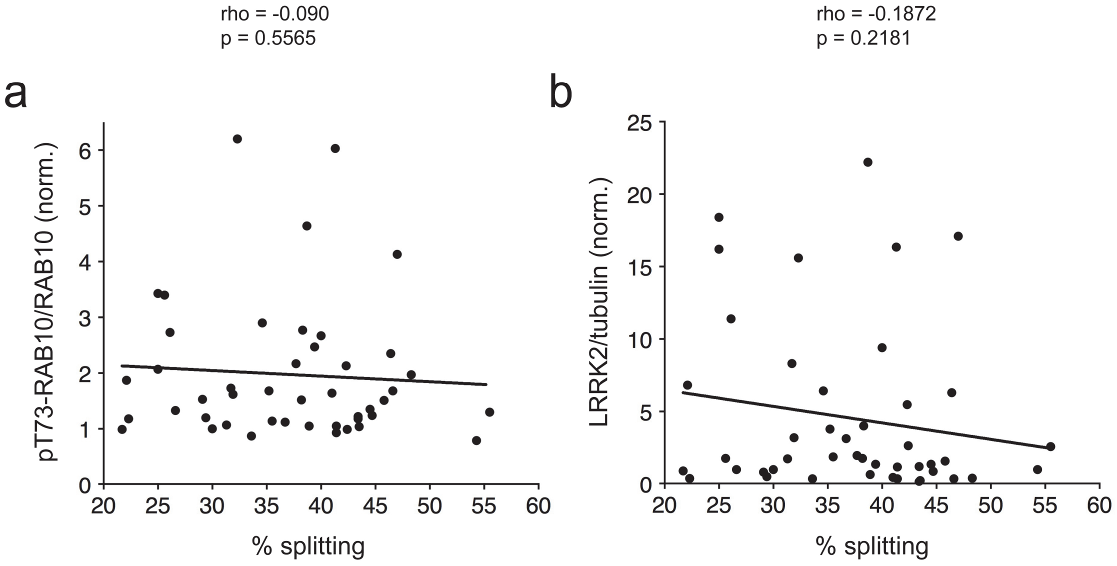


**Supplementary Figure 11. Correlation analysis between pT73-Rab10 or LRRK2 levels and the C/C cohesion phenotype.** (**a**) Spearman correlation analysis between the levels of pT73-Rab10/Rab10 and the C/C cohesion phenotype (% splitting) from all LCL lines analyzed. (**b**) Spearman correlation analysis between the levels of LRRK2/tubulin and the C/C cohesion phenotype (% splitting) from all LCL lines analyzed. Rho and p values are indicated.

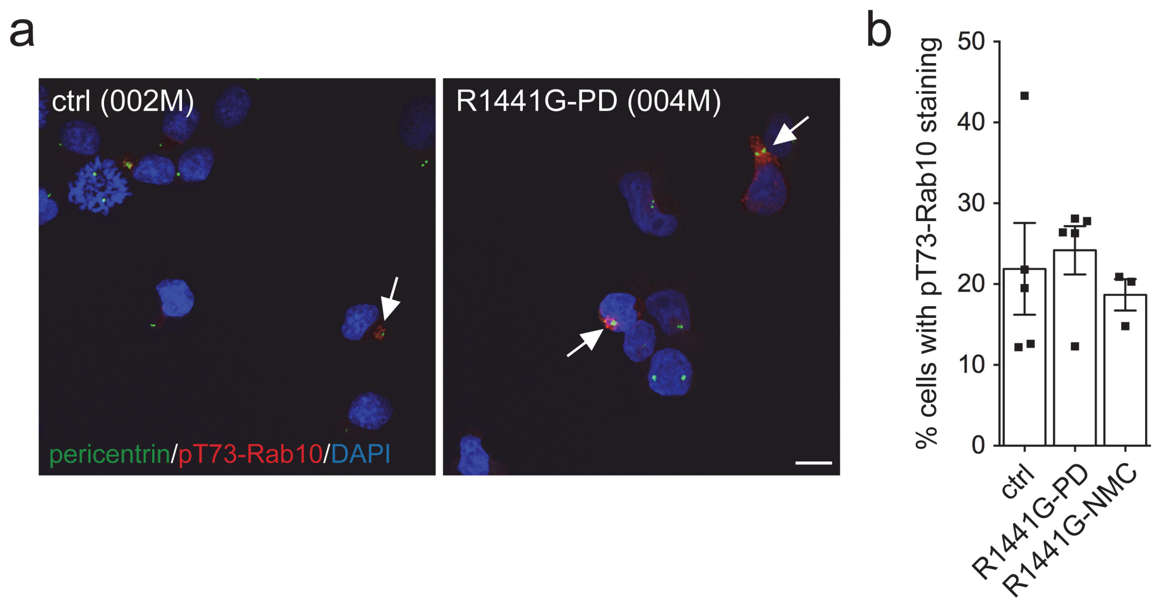


**Supplementary Figure 12. Quantification of the percentage of LCLs displaying pT73-Rab10 staining.** (**a**) Representative images of control (ctrl) and *R1441G-LRRK2* PD LCLs stained with antibodies against pT73-Rab10 (red), pericentrin (green) and DAPI. Arrows point to cells displaying prominent pT73-Rab10 staining. Scale bar, 10 µm. (**b**) Quantification of the percentage of cells displaying pT73-Rab10 staining from 5 control, 5 *R1441G-LRRK2* PD and 3 *R1441G-LRRK2* NMC LCLs. Bars represent mean ± s.e.m.

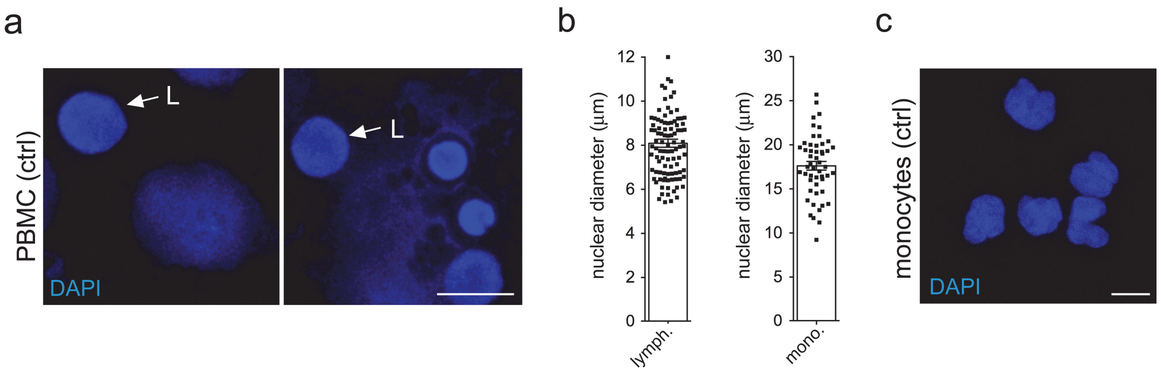


**Supplementary Figure 13. Detection of lymphocytes and monocytes in PBMC preparations.** PBMCs were obtained from the LRRK2 Biobanking Initiative from five healthy controls and five *G2019S-LRRK2* PD patients. PBMCs from the same patients had been purified either employing sodium citrate-coated or heparin-coated tubes and had been cryopreserved at 3 x $10^6$ cells/tube. Cell debris was observed in both preparations, but was particularly extensive in PBMCs purified with sodium citrate-coated tubes. Therefore, analysis was only performed for PBMCs purified with heparin-coated tubes. (**a**) Example of control PBMCs stained with DAPI. L, lymphocytes. Scale bar, 10 µm. (**b**) Average nuclear diameter of lymphocytes (left) (n=98 cells) and monocytes (right) (n=51 cells) from healthy control PBMC preparation as shown in (A). Bars represent mean ± s.e.m. (**c**) Purified monocytes were stained with DAPI. Note the larger and typically kidney-shaped nucleus. Scale bar, 10 µm.

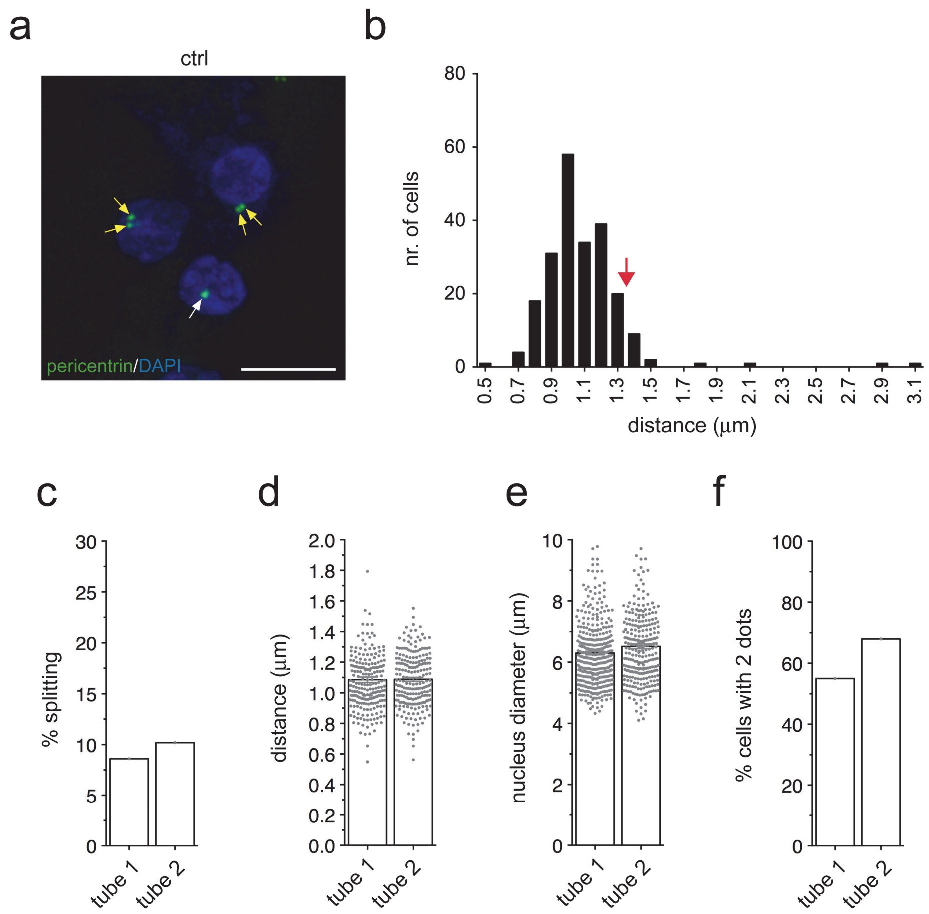


**Supplementary Figure 14. C/C cohesion determination and test-retest reliability in lymphocytes from control PBMC preparation.** (**a**) Example of lymphocytes from healthy control (ctrl) PBMC preparation stained for pericentrin and DAPI. White arrow points to pericentrin-positive structure, yellow arrows point to two pericentrin-positive structures with or without a splitting phenotype. Scale bar, 10 µm. (**b**) Frequency histogram distribution of the number of healthy control lymphocytes displaying two pericentrin-positive dots, with binned distances as indicated. Around 200 cells displaying 2 pericentrin dots were quantified. (**c**) Mean distance between two pericentrin-positive dots. (**d**) Mean nucleus diameter for lymphocytes analyzed. (**e**) Percentage of cells displaying two pericentrin-positive dots. (**f**) Based on the frequency distribution shown in (b), cells were scored as having a split phenotype when the distances between the two pericentrin-positive dots was > 1.3 µm (red arrow), and % splitting is indicated. Two independent cryopreserved PBMC tubes were analyzed one month apart, with excellent test-retest reliability within an individual over time.

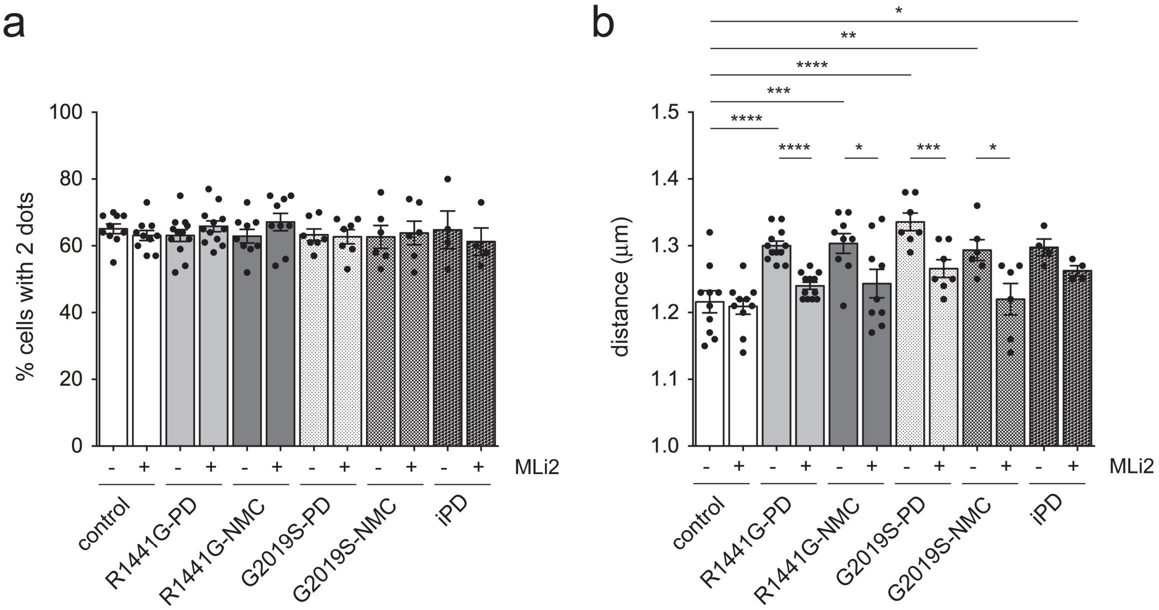


**Supplementary Figure 15. Percentage of cells with two pericentrin-positive dots and mean distances between dots in *R1441G-LRRK2* and *G2019S-LRRK2* lymphocytes.** (**a**) Quantification of the percentage of cells displaying two pericentrin-positive dots from a total of 100-150 lymphocytes per PBMC preparation. Bars represent mean ± s.e.m. (**b**) Mean distance between the two pericentrin-positive dots from lymphocytes of each PBMC preparation. Bars represent mean ± s.e.m.; ctrl versus R1441G mutation ($p < 0.0001$); ctrl versus R1441G NMC ($p = 0.001$); ctrl versus G2019S mutation ($p < 0.0001$); ctrl versus G2019S NMC ($p = 0.007$); ctrl versus idiopathic PD ($p = 0.0125$); R1441G mutation versus R1441G mutation + MLi2 ($p < 0.0001$); R1441G NMC versus R1441G NMC + MLi2 ($p = 0.034$); G2019S mutation versus G2019S mutation + MLi2 ($p = 0.0028$); G2019S NMC versus G2019S NMC + MLi2 ($p = 0.0266$). ****$p < 0.001$; ***$p < 0.005$; **$p < 0.01$; *$p < 0.05$.

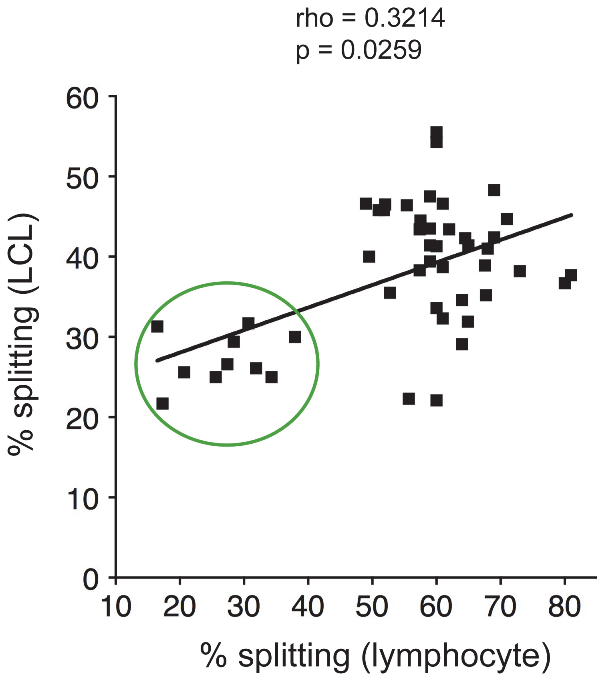


**Supplementary Figure 16. C/C cohesion phenotype in LCLs versus lymphocytes from healthy control and *LRRK2* mutation carriers.** Spearman correlation analysis between the percentage of C/C splitting observed in lymphocytes and in immortalized LCLs from each patient. Note two patients where splitting phenotype was observed in primary lymphocytes but lost upon EBV immortalization. Healthy control patients are circled in green.

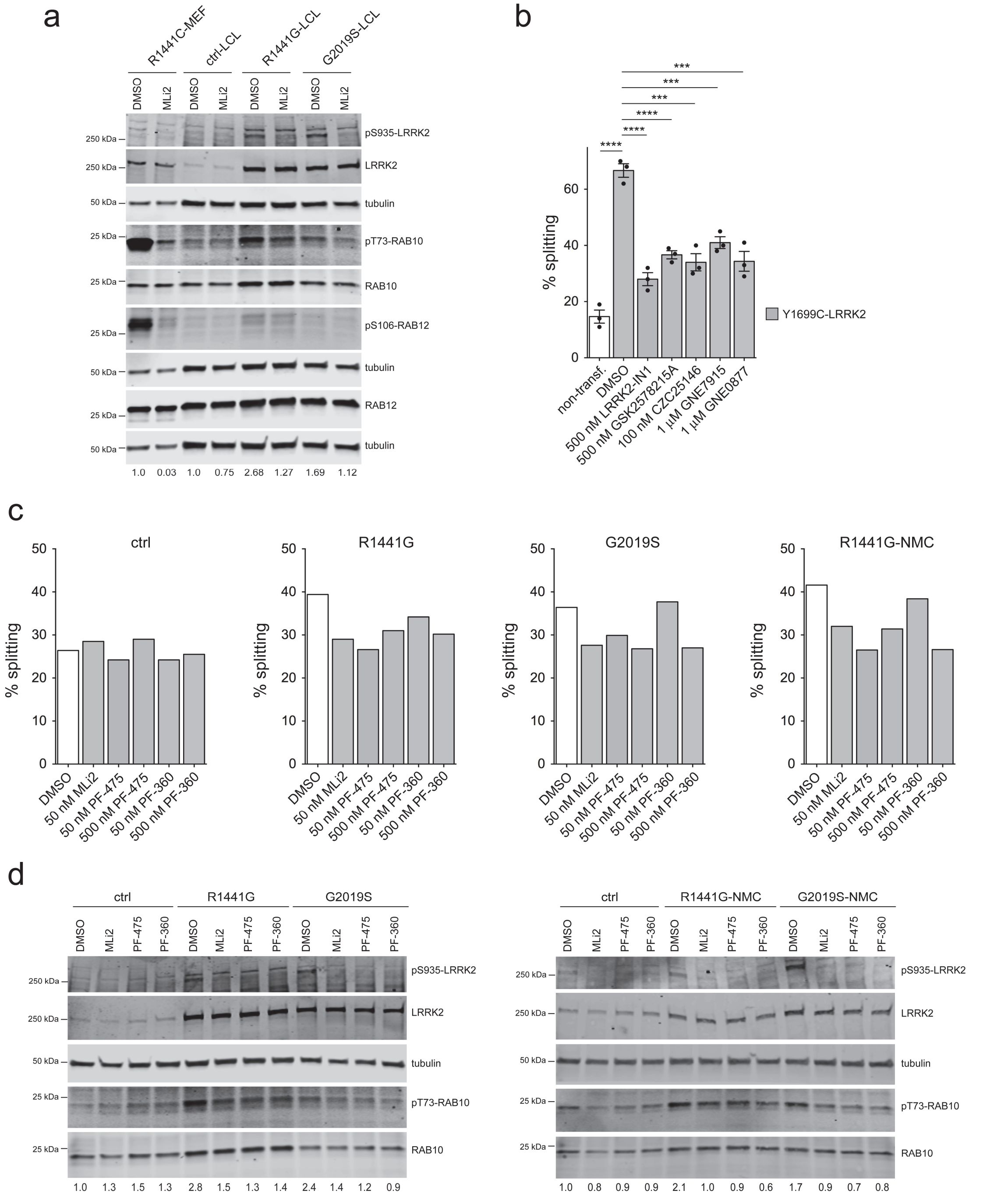


**Supplementary Figure 17. pS106-Rab12 levels in LCLs and effect of distinct LRRK2 kinase inhibitors on the LRRK2-mediated cohesion deficits.** (**a**) R1441C-LRRK2 knockin (KI) MEFs or LCLs were treated with DMSO or MLi2 (500 nM for MEFs, 50 nM for LCLs) for 2 h as indicated, and cell extracts were subjected to multiplexed immunoblot analysis. Values indicate normalized pT73-Rab10/total Rab10

levels for either R1441C-LRRK2-KI MEFs or control LCLs. Note that MLi2-sensitive pT73-Rab10 levels are detectable in both R1441C-LRRK2-KI MEFs and in LCLs, but pS106-Rab12 levels are only detectable in MEFs. (**b**) HEK293T cells were transfected with GFP-tagged Y1699C-LRRK2 and incubated for 2 h with DMSO or various LRRK2 kinase inhibitors before immunocytochemistry using an anti-pericentrin antibody and DAPI. The splitting phenotype was determined from 100 transfected cells per condition and experiment. Bars represent mean ± s.e.m. (N=3 independent experiments; ****$p < 0.001$; ***$p < 0.005$. (**c**) The indicated LCL lines were treated with 50 nM MLi2, 50 nM or 500 nM PF-06447475 (PF-475), or 50 nM or 500 nM PF-360 for 2 h as indicated, and C/C splitting was quantified from around 100-120 cells with two pericentrin dots per condition. (**d**) The indicated LCL lines were treated for 2 h with MLi2 (50 nM), PF-475 (500 nM) or PF-360 (500 nM), and cell extracts were subjected to multiplexed immunoblot analysis. Values indicate pT73-Rab10/total Rab10 levels normalized to control LCL.

| sample ID | sequencing |
|---|---|
| S004 | |
| S005 | |
| S006 | |
| S008 | |
| S009 | |
| S007 | |
| S010 | |
| S011 | |
| S012 | GIGYF2 P1219_Q1222de (rs763368000) |
| S013 | |
| S021 | PRKN R376W heterozygote (rs34424986) |
| S022 | |
| S023 | |
| S024 | |
| S025 | |
| S026 | |
| S027 | |
| S028 | |
| S029 | ATP13A2 p.Y976Tfs* heterozygote (rs765632065) |
| S014 | |
| S015 | |
| S016 | |
| S017 | |
| S018 | |
| S019 | |
| S020 | GBA E326K (rs2230288) |
| S030 | |
| S031 | GBA I158 synonymous (rs147411159) |
| S032 | |
| S033 | |
| S034 | |
| S035 | PRKN R376W homozygote (rs34424986) |
| S036 | |
| S037 | GBA E326K (rs2230288) + T369M (rs75548401) |
| S038 | |

| Major PD genes | |
|---|---|
| ATP13A2 | Parkinson´s Disease |
| CHCHD2 | Parkinson´s Disease |
| FBXO7 | Parkinson´s Disease |
| HTRA2 | Parkinson´s Disease |
| LRRK2 | Parkinson´s Disease |
| NR4A1 | Parkinson´s Disease |
| PARK2=PRKN | Parkinson´s Disease |
| PARK7 | Parkinson´s Disease |
| PINK1 | Parkinson´s Disease |
| RAB39B | Parkinson´s Disease |
| SNCA | Parkinson´s Disease |
| UCHL1 | Parkinson´s Disease |
| VPS13C | Parkinson´s Disease |
| VPS35 | Parkinson´s Disease |
| PLA2G6 | Parkinson´s Disease |
| GIGYF2 | Parkinson´s Disease/Dementia |
| PLA2G6 | Parkinsonism |

**Supplementary Table 1. Several LCL lines display PD-relevant mutations.** All 35 LCL PD lines were subjected to whole exome sequencing and long-range PCR and Sanger sequencing of the GBA gene, the most frequent known genetic risk factor for PD. The 10 PD LCL lines with a cohesion phenotype are boxed in red. Apart from GBA, the major PD genes analyzed are indicated to the right; de, deletion; fs, frameshift.

**Supplementary Table 2. Results summary of TRAPD gene/protein burden analysis.**

| Model | Genes (p<0.05) | Protein Domains (p<0.05) | Gene names | Top Gene Ontology (PANTHER) |
|---|---|---|---|---|
| Dominant - Gene | 31 | n/a | ABR<br>ADAMTS10<br>ANKRD20A1<br>ARL9<br>BTN3A2<br>CBWD3<br>CDC7<br>CNOT2<br>DNAH9<br>FBXO46<br>GOLGA8N<br>GPC2<br>IVL<br>KLHL33<br>KRTAP4-9<br>LOC100129697<br>LOC101059915<br>MUC20<br>NIPA1<br>NOTCH2NLC<br>RNF19B<br>SCN4A<br>TBC1D3D<br>TBC1D3F<br>THAP11<br>TSHZ1<br>UNC80<br>WASHC2C<br>ZNF343<br>ZNF705B<br>ZSWIM6 | Activation of GTPase activity ($p=2.94x10^{-5}$,FDR=0.47) |
| Dominant – Protein Domain | 58 | 220 | ADAMTS10<br>ALMS1<br>ANKRD20A1<br>AR<br>ARL9<br>BTN3A2<br>CBWD1<br>CBWD3<br>CCDC187<br>CDC7<br>CNOT2<br>DENND4B<br>DNAH12<br>DNAH9<br>ERVW-1<br>FAM120B<br>FAM205C<br>FBXO46<br>GOLGA8N<br>GPC2<br>HLA-DPB1<br>IVD<br>IVL<br>KCNH2<br>KIR2DL4<br>KIR2DS4<br>KIR3DL1<br>KLHL33<br>KRTAP4-9<br>LOC100129697<br>LOC101059915<br>MADCAM1<br>MUC20 | Activation of GTPase activity ($p=3.73x10^{-4}$,FDR=1) |

| | | | | |
|---|---|---|---|---|
| | | | MUC4<br>NBPF10<br>NBPF12<br>NBPF14<br>NBPF9<br>NIPA1<br>NOTCH2NLC<br>PDE4DIP<br>PLIN4<br>POTEB3<br>PRAMEF13<br>PRAMEF15<br>RNF19B<br>SCN4A<br>SPEM3<br>TBC1D3B<br>TBC1D3D<br>TBC1D3F<br>THAP11<br>TSHZ1<br>UNC80<br>WASHC2C<br>ZNF343<br>ZNF705B<br>ZSWIM6 | |
| Recessive - Gene | 6 | n/a | FAM120B<br>NOTCH2NLC<br>PDZD4<br>TBC1D3D<br>TSHZ1<br>ZNF705B | Activation of GTPase activity ($p=6.59x10^{-4}$,FDR=1) |
| Recessive - Protein Domain | 11 | 30 | AR<br>FAM120B<br>KIR2DS4<br>KIR3DL1<br>KLHL33<br>NBPF10<br>NOTCH2NLC<br>PDZD4<br>TBC1D3D<br>TSHZ1<br>ZNF705B | Androgen receptor signaling pathway ($p=7.66x10^{-5}$,FDR=1) |

| sample ID | TBC1D3D | NOTCH2NLC | DUF1220 | combined |
|---|---|---|---|---|
| S026 | 0 | 0 | 0 | 0 |
| S015 | 0 | 0 | 0 | 0 |
| S014 | 0 | 0 | 0 | 0 |
| S035 | 0 | 0 | 0 | 0 |
| S029 | 0 | 0 | 1 | 1 |
| S028 | 0 | 0 | 1 | 1 |
| S024 | 0 | 0 | 1 | 1 |
| S027 | 0 | 0 | 1 | 1 |
| S022 | 0 | 0 | 1 | 1 |
| S033 | 0 | 0 | 1 | 1 |
| S001 | 0 | 0 | 1 | 1 |
| S032 | 0 | 0 | 1 | 1 |
| S031 | 0 | 0 | 1 | 1 |
| S025 | 0 | 0 | 1 | 1 |
| S016 | 0 | 0 | 1 | 1 |
| S019 | 0 | 0 | 2 | 2 |
| S021 | 0 | 0 | 2 | 2 |
| S017 | 0 | 0 | 2 | 2 |
| S018 | 0 | 0 | 2 | 2 |
| S030 | 0 | 0 | 2 | 2 |
| S020 | 0 | 0 | 2 | 2 |
| S037 | 0 | 0 | 3 | 3 |
| S036 | 0 | 0 | 3 | 3 |
| S034 | 0 | 0 | 3 | 3 |
| S023 | 0 | 2 | 1 | 3 |
| S038 | 0 | 2 | 2 | 4 |
| *mean* | *0* | *0.153* | *1.346* | *1.5* |
| S012* | 0 | 0 | 0 | 0 |
| S007 | 0 | 0 | 4 | 4 |
| S013 | 0 | 0 | 4 | 4 |
| S009 | 0 | 0 | 4 | 4 |
| S006 | 1 | 0 | 3 | 4 |
| S010 | 0 | 2 | 3 | 5 |
| S008 | 2 | 2 | 2 | 6 |
| S004 | 0 | 2 | 5 | 7 |
| S011 | 2 | 0 | 5 | 7 |
| S005 | 2 | 2 | 6 | 10 |
| *mean* | *0.7* | *0.8* | *3.6* | *5.1* |

**Supplementary Table 3. Gene burden analysis for rare variants employing whole exome sequencing data from the different PD LCL lines.** Whilst there is no significant burden of rare variants which correlates with the centrosomal cohesion phenotype for any single gene after correcting for multiple comparisons, there is an association between the centrosome phenotype and rare variants within the *TBC1D3D* gene and the *NOTCH2NLC* gene. Pathway analysis also indicates enrichment for a list of genes enriched for the DUF1220 domain. Numbers indicate the number of rare variants identified for each LCL line. The 10 PD LCL lines with a cohesion phenotype are boxed in red. *Note that S012 is the line containing a mutation in *GIGYF2*.
