# Supplementary material for: A potential patient stratification biomarker for Parkinson´s disease based on LRRK2 kinase-mediated centrosomal alterations in peripheral blood-derived cells
Source: NPJ Parkinsons Dis. 2024 Jan 8;10:12. doi: 10.1038/s41531-023-00624-8 (PMC10774440; doi:10.1038/s41531-023-00624-8)
Supplement: Supplementary file 2 — reporting summary [file 41531_2023_624_MOESM2_ESM.pdf]

nature portfolio

Corresponding author(s): Sabine Hilfiker, PhD

Last updated by author(s): Dec 10, 2023

Reporting Summary

Nature Portfolio wishes to improve the reproducibility of the work that we publish. This form provides structure for consistency and transparency in reporting. For further information on Nature Portfolio policies, see our Editorial Policies and the Editorial Policy Checklist.

Statistics

For all statistical analyses, confirm that the following items are present in the figure legend, table legend, main text, or Methods section.

n/a	Confirmed	
☐	☒	The exact sample size (*n*) for each experimental group/condition, given as a discrete number and unit of measurement
☐	☒	A statement on whether measurements were taken from distinct samples or whether the same sample was measured repeatedly
☐	☒	The statistical test(s) used AND whether they are one- or two-sided<br>*Only common tests should be described solely by name; describe more complex techniques in the Methods section.*
☐	☒	A description of all covariates tested
☐	☒	A description of any assumptions or corrections, such as tests of normality and adjustment for multiple comparisons
☐	☒	A full description of the statistical parameters including central tendency (e.g. means) or other basic estimates (e.g. regression coefficient) AND variation (e.g. standard deviation) or associated estimates of uncertainty (e.g. confidence intervals)
☐	☒	For null hypothesis testing, the test statistic (e.g. *F*, *t*, *r*) with confidence intervals, effect sizes, degrees of freedom and *P* value noted<br>*Give P values as exact values whenever suitable.*
☒	☐	For Bayesian analysis, information on the choice of priors and Markov chain Monte Carlo settings
☒	☐	For hierarchical and complex designs, identification of the appropriate level for tests and full reporting of outcomes
☐	☒	Estimates of effect sizes (e.g. Cohen's *d*, Pearson's *r*), indicating how they were calculated

Our web collection on statistics for biologists contains articles on many of the points above.

Software and code

Policy information about availability of computer code

Data collection	No software was used.
Data analysis	Statistical analysis was performed using Prism software version 9.5 (GraphPad, San Diego, CA).

For manuscripts utilizing custom algorithms or software that are central to the research but not yet described in published literature, software must be made available to editors and reviewers. We strongly encourage code deposition in a community repository (e.g. GitHub). See the Nature Portfolio guidelines for submitting code & software for further information.

Data

Policy information about availability of data

All manuscripts must include a data availability statement. This statement should provide the following information, where applicable:

- Accession codes, unique identifiers, or web links for publicly available datasets
- A description of any restrictions on data availability
- For clinical datasets or third party data, please ensure that the statement adheres to our policy

Transparency and reproducibility: the de-identified genetic data (whole exome sequence variant call file (VCF)) and their associated phenotype data (MLi2-sensitive cohesion phenotype) are available in Zenodo (DOI 10.5281/zenodo.1027821). Raw Western blot data and all sequencing data are available as supplemental figures and tables. All raw images of cohesion determinations are available upon request.

Research involving human participants, their data, or biological material

Policy information about studies with human participants or human data. See also policy information about sex, gender (identity/presentation), and sexual orientation and race, ethnicity and racism.

Reporting on sex and gender	To account for potential sex-specific differences as reported for the prevalence of sporadic Parkinson's disease, healthy controls, idiopathic Parkinson's disease patients, Parkinson's disease patients with LRRK2 mutations or non-manifesting LRRK2 mutation carriers were chosen to represent both sexes.
Reporting on race, ethnicity, or other socially relevant groupings	To account for differences in the prevalence of LRRK2-related Parkinson's disease across distinct populations, healthy control and PD patients/LRRK2 mutation carriers were of similar self-reported race/ethnicity.
Population characteristics	Participants were of similar age and both sexes, and PD patients harbouring distinct LRRK2 mutations were chosen to be of similar age at diagnosis, disease duration and disease severity.
Recruitment	Participants were recruited with written informed consent and examined by neurologists specialized in movement disorders. Subjects donated blood samples for genotyping and peripheral blood-derived cells for direct cell biological analysis. None of the controls had a family history of Parkinson's disease, and all control and idiopathic Parkinson's disease samples were negative for the G2019S-LRRK2 or R1441G-LRRK2 mutation.
Ethics oversight	The study protocols were approved by the University of New South Wales human research ethics committee, the Donostia University Hospital in San Sebastian ethics committee, and the IRBs of both Columbia University (CUIMC).

Note that full information on the approval of the study protocol must also be provided in the manuscript.

Field-specific reporting

Please select the one below that is the best fit for your research. If you are not sure, read the appropriate sections before making your selection.

☒ Life sciences ☐ Behavioural & social sciences ☐ Ecological, evolutionary & environmental sciences

For a reference copy of the document with all sections, see nature.com/documents/nr-reporting-summary-flat.pdf

Life sciences study design

All studies must disclose on these points even when the disclosure is negative.

Sample size	Sample size was chosen based on previously determined effect sizes and power analysis.
Data exclusions	No data were excluded.
Replication	In some cases, images were independently quantified by an additional observer blind to condition and at distinct research sites, with identical results obtained in all cases. For each LCL line, fresh extracts from 2-3 independent cultures were analyzed, with similar results obtained in all cases.
Randomization	Samples were allocated into experimental groups based on clinical phenotype and genetic mutation status.
Blinding	Investigators were blinded to genotype and phenotype during image acquisition and analysis as well as for the western blotting experiments.

Reporting for specific materials, systems and methods

We require information from authors about some types of materials, experimental systems and methods used in many studies. Here, indicate whether each material, system or method listed is relevant to your study. If you are not sure if a list item applies to your research, read the appropriate section before selecting a response.

Materials & experimental systems

n/a	Involved in the study	
☐	☒	Antibodies
☐	☒	Eukaryotic cell lines
☒	☐	Palaeontology and archaeology
☒	☐	Animals and other organisms
☒	☐	Clinical data
☒	☐	Dual use research of concern
☒	☐	Plants

Methods

n/a	Involved in the study	
☒	☐	ChIP-seq
☒	☐	Flow cytometry
☒	☐	MRI-based neuroimaging

Antibodies

Antibodies used	For immunocytochemistry, primary antibodies included mouse monoclonal anti-gamma-tubulin (1:1000, Abcam ab11316), mouse monoclonal anti-LAMP1 (1:500, Santa Cruz Biotechnology, sc-20011), rabbit polyclonal anti-pericentrin (1:1000, Abcam ab4448) and rabbit monoclonal anti-pT73-Rab10 (1:1000, Abcam ab241060).<br>For Western blotting, primary antibodies included rabbit anti-S935-LRRK2 antibody (1:500, Abcam, ab133450), mouse monoclonal anti-LRRK2 antibody (1:1000, Antibodies Inc, 75-235), mouse monoclonal anti-gamma-tubulin antibody (1:10´000, Sigma, clone DM1A), rabbit monoclonal anti-pT73-Rab10 antibody (1:1000, Abcam, ab230261), mouse monoclonal total Rab10 antibody (1:1000, Sigma, SAB5300028), rabbit monoclonal anti-pS106-Rab12 antibody (1:1000, Abcam, ab256487) or a rabbit polyclonal total Rab12 antibody (1:500, ProteinTech, 18843-1-AP). In most cases, antibodies had been commercially validated against the respective knockout cell extracts.
Validation	In most cases, antibodies have been commercially validated against their respective knockout cell extracts.

Eukaryotic cell lines

Policy information about cell lines and Sex and Gender in Research

Cell line source(s)	PBMCs from human participants, EBV-immortalized lymphocytes from human participants, in both cases from both sexes. HEK293T cells (human embryonic kidney cell line, derived from female fetus). Murine embryonic fibroblast cells from R1441C-LRRK2 knockin mice (both sexes).
Authentication	Identity of the HEK293T cell line was assessed by short tandem repeat profiling. Given the vastly different morphologies of the distinct human cell types employed, their morphology was also inspected on a regular basis to assure purity.
Mycoplasma contamination	All cell lines underwent monthly mycoplasma testing.
Commonly misidentified lines<br>(See ICLAC register)	*Name any commonly misidentified cell lines used in the study and provide a rationale for their use.*
